# Three-dimensional analysis of intraepidermal nerve fibres and Langerhans cells in keloids with a focus on pruritus

**DOI:** 10.1038/s41598-025-11674-8

**Published:** 2025-08-19

**Authors:** Haruka Matsuzoe, Koh-ei Toyoshima, Miki Takase, Ayako Tsuchiya, Mika Ikeda, Motoko Naitoh, Kazuya Kataoka, Tomoya Kawabata, Miho Ogawa, Naoki Morimoto, Takashi Tsuji

**Affiliations:** 1 Department of Plastic and Reconstructive Surgery, Graduate School of Medicine, Kyoto University, Kyoto, 606-8507 Japan; 2 Laboratory for Organ Regeneration, RIKEN Center for Biosystems Dynamics Research (BDR), Kobe, 650-0047 Japan; 3 OrganTech Inc., Tokyo, 104-0053 Japan; 4 TechnoPro, Inc., Tokyo, 106-6135 Japan; 5 Department of Plastic and Reconstructive Surgery, Kobe City Medical Center General Hospital, Kobe, 650-0047 Japan

**Keywords:** Keloids, Pruritus, Intraepidermal nerve fibres, Langerhans cells, Signs and symptoms, Pruritus

## Abstract

Keloids are intractable dermato-fibrotic lesions that progressively expands from the primary lesion to the surrounding normal areas. In this study, we investigated uncontrollable pruritus in keloids and the association between keloid symptoms and pruritus using three-dimensional immunofluorescence for the analysis of nerve fibres and Langerhans cells in the anterior chest and ear keloids, and the analysis of messenger-RNA expression of substance P as a pruritus mediator. Nerve fibres infiltrating the epidermis were numerous in the periphery of the anterior chest keloids, where pruritus was reported, with some extending into the granular layer. However, there was no difference in Langerhans cells during pruritus, location or region of the keloid. Furthermore, we observed substance P gene expression in the periphery of the anterior chest keloids. Our observational study suggests an association between intractable pruritus in anterior keloid lesions, increased intraepidermal nerve fibre density, and elevated expression of Th2 cytokines and substance P.

**Supplementary Information:**

The online version contains supplementary material available at 10.1038/s41598-025-11674-8.

## Introduction

Keloids are fibrotic lesions arising from abnormal wound healing. They can develop from minor skin injuries, including insect bites or acne. Keloids are benign; however, they can expand into the surrounding normal skin through the rapid proliferation of fibroblast-like cells^1^. The clinical features include induration with erythema, elevation, erythematous infiltration into the surrounding area, pruritus, and pain. Treatment at specialised facilities is recommended for these conditions. The treatment includes surgical excision accompanied by postoperative irradiation, application or local injection of steroids, and oral tranilast administration, which inhibits the release of chemical mediators. Persistent and intractable pruritus is frequently observed even after apparent clinical improvement with various therapeutic interventions, significantly impairing patients’ quality of life^2,3^. Notably, keloids located on the anterior chest are subject to high mechanical tension and exhibit a high rate of recurrence, thereby warranting particularly cautious surgical management^4^. Keloids are unique to humans, and their pathophysiology remains unclear; therefore, developing fundamental treatment approaches is difficult. Elucidating the pathogenesis of pruritus in keloids may lead to the discovery of effective therapeutic targets for intractable pruritus^5,6^.

Various reports have described the cellular and molecular mechanisms underlying pruritus in keloids. Histological studies have shown that keloids exhibit an increase in mast cells, macrophages, lymphocytes, and neutrophils, and elevated levels of Th2 cytokines, including interleukin-4 (IL-4) and IL-13, which are implicated in pruritus; thereby suggesting a potential association with the exacerbation of pruritus^3,7–10^. Other studies have focused on nerve function abnormalities within keloids. Nerve fibres in keloids are compromised by their secondary entrapment due to excessive collagen deposition, leading to afferent nociceptive neuron damage, similar to compressive neuropathy. Consequently, regenerated C-fibres are believed to transmit pruritus^11^. Furthermore, damaged nerve fibres release neuropeptides such as substance P (SP), which mediates neurogenic inflammation and contributes to the onset of pruritus^11–16^. The mechanism of pruritus in keloids is multifactorial, and there have been reports on the efficacy of various treatment modalities. However, there is no consensus on the treatment of pruritus in keloids^5^. Consequently, the treatment is challenging, therefore highlighting the urgent need to elucidate the underlying pathophysiology^17^.

Pruritus is defined as an unpleasant sensation that triggers the urge to pruritus and a benign physiological sensation prompting the physical removal of foreign substances in response to various external stimuli. However, if severe or persists chronically (> 6 weeks), it can decrease the quality of life significantly, with the need for urgent treatment^6,18^. Diseases associated with intense pruritus comprise inflammatory skin diseases such as atopic dermatitis (AD) and psoriasis. Studies on these conditions have reported on the distribution and number of intraepidermal nerve fibres, indicating that the distribution and density of these nerve fibres within the epidermis may be involved in severe pruritus^19,20^. Therefore, pruritus is transmitted through pruritus-selective unmyelinated C-fibres in the epidermis and dermoepidermal junctions. These fibres include both histamine- and non-histamine-sensitive neurons. Neurogenic inflammation involving non-histamine-sensitive neurons is associated with inflammatory skin conditions, such as AD, which is characterised by chronic pruritus. Pruritus mediators corresponding to non-histamine-sensitive neurons have garnered attention. These pruritus mediators stimulate the nerves directly to induce pruritus, thereby serving as therapeutic targets for intractable pruritus; new treatments have been developed accordingly^6,21–24^. In addition, there is an increased proliferation of Langerhans cells in the epidermis of patients with AD. These cells promote a Th2-dominant state, indicating their contribution to AD development^25–27^.

In this study, we aimed to investigate pruritus in keloids by focusing on its regional manifestation, conducted a three-dimensional analysis of the distribution of nerve fibres and Langerhans cells within the keloid epidermis. Anterior chest keloids are constantly exposed to mechanical stress and are frequently associated with intense pruritus, whereas most ear keloids don’t exhibit pruritus^28^. Therefore, we compared anterior chest keloids with ear keloids. We successfully found differences in the distribution of damaged skin appendages with inflammatory cell infiltration, morphological characteristics of the epidermal layer, and penetration of nerve fibres into the epidermal layer between the two types of keloid tissues. Furthermore, we demonstrated that SP was expressed at the boundary between keloid and normal tissue in the anterior chest. These results indicated that the pathology of keloids can be classified into two pathogenic types and by inhibiting or blocking inflammatory mediators suggested the possibility of the clinical application in the treatment of keloid-related pruritus.

## Results

### Characteristic analysis of the clinical findings in cases of keloid

We compared anterior chest and ear keloids with normal abdominal skin to clarify the clinical characteristics in the cases of keloids. Six patients (2 and 4 females and males, respectively) and 6 patients (5 and 1 females and male, respectively) who were 36 to 69 and 22 to 32 years old (mean 47.6 and 26.3 years old, respectively) constituted the cases of the anterior chest and ear keloids (Table 1). Normal abdominal skin as a control group was obtained from three female patients aged 43 to 52 years old (mean 49 years old) with malignant tumours or burn. The normal skin was received from patients who had undergone resection of malignant tumours and subsequently required tissue reconstruction using a rectus abdominis myocutaneous flap, or from burn patients’ abdominal skin following skin grafting (Table 1). All cases of the anterior chest and ear keloids were scored 15 or higher and under 15, respectively, following the analyses of the clinical findings of each patient using the Japan Scar Workshop Scar Scale (JSS)^29^. Keloid development originated from acne or surgical scars and erythematous infiltration around the sites, with complaints of intense pruritus found in those areas in all patients in the anterior chest keloid group. All cases in the ear keloid group were initiated by piercing; however, intense pruritus was not observed (Table 1). The pruritus scoring was assessed using the evaluation table of JSS. All patients in the anterior chest keloid group scored 3 points, indicating severe pruritus, whereas all patients in the ear keloid group scored 0 points, indicating no pruritus (Table 1).

**Table 1:** Comparison of clinical findings in keloid cases.

Specimen	Site	ID	JSS (PS)	Age (years)	Sex	Causes	Evaluation					Sample types
							Induration	Elevation	Redness	Erythema around scars	Pruritus	
Normal skin	Abdomen	N1	–	52	F	–	–	–	−	−	−	PP/PFr
		N2	–	52	F	–	–	–	−	−	−	PP/PFr
		N3	–	43	F	–	–	–	−	−	−	PP/PFr
Keloid	Anterior chest	KA1	17(3)	69	M	P	+	+	++	+	+	PP
		KA2	16(3)	40	M	A	+	+	++	+	+	PP
		KA3	20(3)	36	F	A	+	+	++	+	+	PP/PFr
		KA4	22(3)	49	M	P	+	+	++	+	+	PP/PFr*
		KA5	17(3)	42	M	P	+	+	++	+	+	PP/PFr
		KA6	17(3)	50	F	P	+	+	++	+	+	PP/PFr
	Ear	KE1	10(0)	32	M	Pi	+	++	++	+	−	PP
		KE2	10(0)	29	F	Pi	+	++	+	−	−	PP
		KE3	13(0)	22	F	Pi	+	++	+	−	−	PP/PFr
		KE4	13(0)	23	F	Pi	+	++	+	−	−	PP/PFr
		KE5	8(0)	28	F	Pi	+	++	+	−	−	PP/PFr*
		KE6	11(0)	24	F	Pi	+	++	+	−	−	PP/PFr

N, Normal skin; KA, Anterior chest wall keloids; KE, Ear keloids. JSW, Japan Scar Workshop (JSW) Scar Scale; PS, Pruritus scoring. A, Acne; P, Postoperative; Pi, Pierce. PP, 4% paraformaldehyde-fixed paraffin-embedded; PFr, 4% paraformaldehyde-fixed frozen. *, Due to the small number of samples, these were not suitable for analysis.

**Fig. 1:**
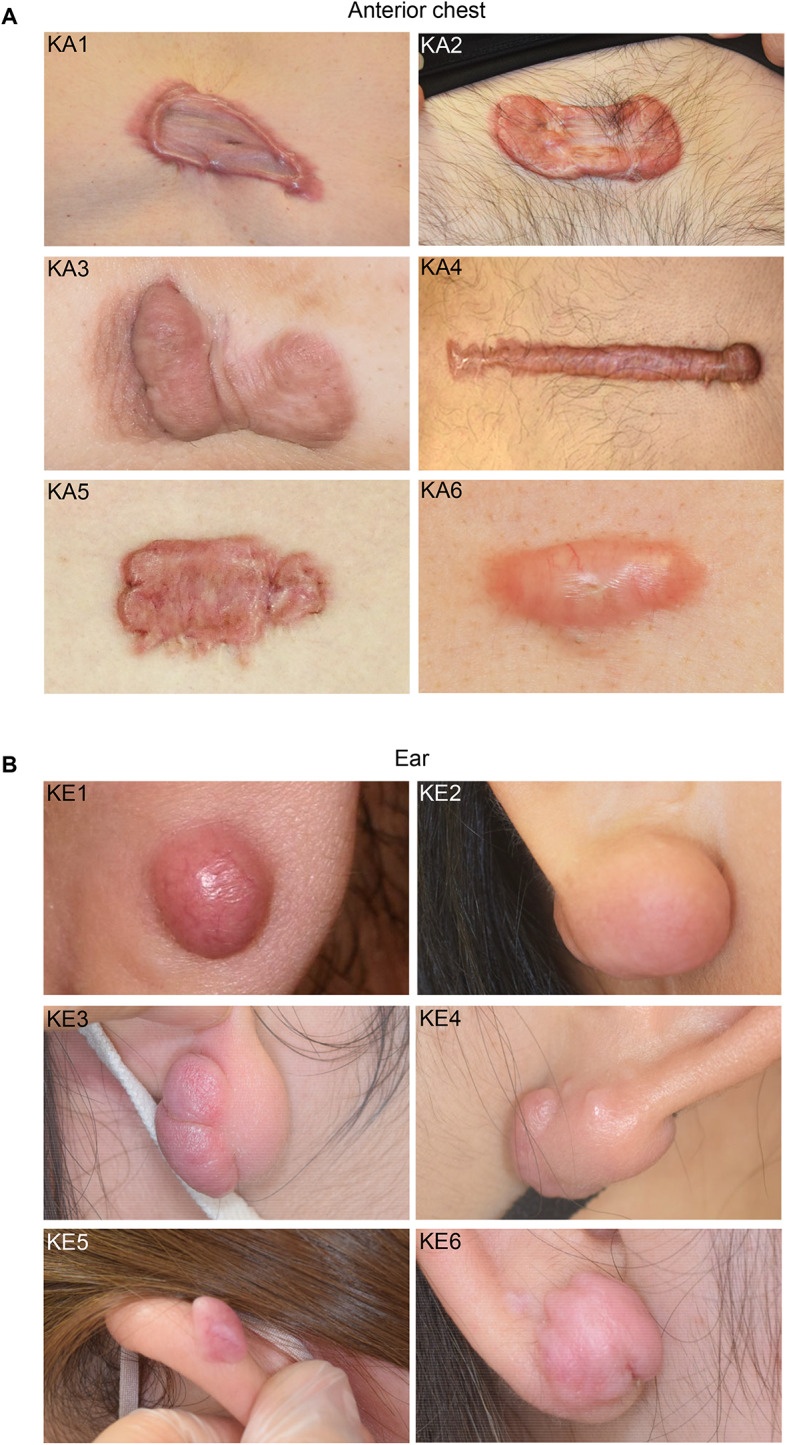
Macroscopic analysis of keloids on the anterior chest and ear. (**A**) Photographs of anterior chest keloids. (**B**) Photographs of ear keloids. Case IDs are indicated in the upper left corner of each photograph.

Macroscopic examination of the anterior chest keloids revealed irregular superficial spread with significant erythema, particularly at the edges and surrounding erythematous infiltrates (Fig. 1A). Some anterior chest keloids showed highly elevated edges with severe erythema, whereas the central areas were flat with mild erythema (Fig. 1A, KA1, 2, and 5). In contrast, ear keloids displayed a regular round shape with well-defined borders that expanded in a bulging manner (Fig. 1B). The erythema in ear keloids was predominantly mild in five of six cases, compared to the marked erythema in anterior chest keloids, with surrounding erythematous infiltration present in only one of six cases (Fig. 1B, KE1). These clinical findings indicated that both groups of keloids, occurring at different sites, had similar primary findings, including induration, elevation, and redness, and can be distinguished by the erythema and pruritus around the lesion.

### Comparative analysis between anterior chest and ear keloids using immunohistochemistry

**Fig. 2:**
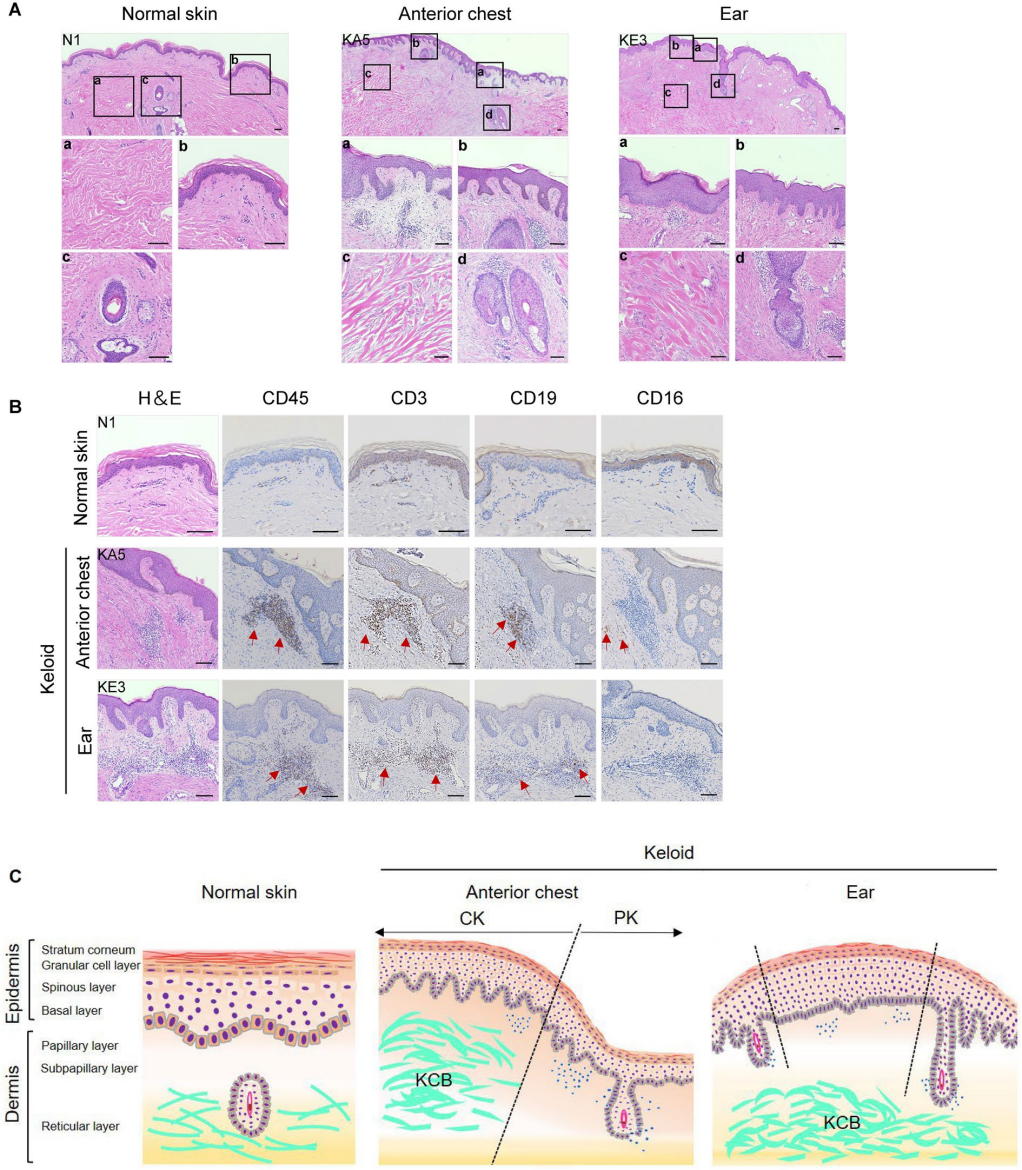
Regional specificity of keloid formation in various anatomical sites. (**A**) H&E staining of normal skin, anterior chest and ear keloids. Low magnification images of each tissue (top) and high magnification images (a, b, c and d) of each boxed area are shown. Alphabets in boxes of low magnification images correspond to high magnification images. Scale bars, 100 μm. (**B**) Immunohistochemical staining of CD45, CD3, CD19 and CD16 in normal skin, anterior chest and ear keloids. Dark brown dots (The red arrows) indicate positively stained cells. Scale bars, 100 μm. (**C**) Schematic representation of anterior chest and ear keloids and normal skin tissue. The black dotted line is the boundary in the regional classification of keloids. CK, Central region of the keloid; PK, Peripheral region of the keloid; KCB, Keloid collagen bundles.

We initially compared the histology between normal skin and the two keloid types. The stratum corneum of the normal skin appeared as parallel layers of uniform thickness, whereas that of both keloid types showed irregular and thin layers and lacked attachment to the epithelia (Fig. 2A and Supplementary Fig. S1). Both types of keloids showed thickening of the epidermal layer; however, rete ridges developed throughout ​the anterior chest keloid area and were localised in the ear keloids (Fig. 2A and Supplementary Fig. S1).

We found disorganised, thickened, hyalinised, and eosinophilic collagen bundles^30^ in the reticular dermis of both keloids (Fig. 2A, KA5 and KE3, c and Supplementary Fig. S1). The other hand, in the boundary region between the lesion of anterior chest keloid and normal skin, it was shown that the destruction of hair follicles accompanied by infiltration of inflammatory cells (Fig. 2A, KA5, d and Supplementary Fig. S1). The inflammatory cell infiltration around the skin appendages in the ear keloids was milder than that in the anterior chest keloids and was widely distributed in the keloidal tissue (Fig. 2A, KE3, d and Supplementary Fig. S1). We immunostained the specimens using anti-CD45, CD3, CD19, and CD16 antibodies to analyse the inflammatory cells in the keloids. These inflammatory cells expressed CD45, CD3, and CD19 antibodies and were immuno-negative for anti-CD16 antibodies (Fig. 2B). Cells expressing CD16 antibodies were found in the vicinity of clusters of inflammatory cells. These results indicate that inflammatory cells in keloids are composed of T cells and B cells, with T cells predominating. We also show that CD16-positive cells are scattered around lymphocytic infiltrates. However, these results are based on qualitative rather than quantitative analysis.

Histological findings showed clear differences between the anterior chest and ear keloids regarding the morphology and distribution of epidermal layer thickening and rete ridge, location of skin appendage destruction, and distribution of inflammatory cell infiltration (Table 2 and Fig. 2C). Therefore, we observed both macroscopic and histological differences between the anterior chest and ear keloids. Anterior chest keloids are characterised by pruritic areas with corresponding erythematous infiltrates in the surrounding area, and the results of histochemical analysis of inflammatory cell infiltrates and destruction of skin appendages are consistent with this finding. We suggested regional specificity in keloid formation.

**Table 2:** Comparison of histological findings in keloid cases.

Site	ID	Phenotype	Epithelium	Skin appendages	Inflammatory cell infiltration
Anterior chest	KA1	I	C^1^C^2^	LB_HF_	SD&SA
	KA2	I	A	LB_HF_	SD&SA
	KA3	I	A	LB_HF_	SD&SA
	KA4	I	C^1^C^2^	LB_SG_	SD&SA
	KA5	I	A	LB_HF_	SD&SA
	KA6	I	A	LB_HF_	SD&SA
Ear	KE1	II	C^1^C^2^	NL_HF_	SD
	KE2	II	C^1^C^2^	NL_HF&SG_	SD
	KE3	II	C^1^C^2^	NL_HF_	SD
	KE4	II	C^1^C^2^	NL_HF_	SD
	KE5	II	C^1^C^2^	NL_HF_	SD&SA
	KE6	II	C^1^C^2^	NL_HF_	SD

Phenotype: I, Spreading; II, Bulging.

Epithelium: A, Continuity and regular elongation of rete ridges; B, Continuity and epidermal thickening; C^1^Discontinuity and regular elongation of rete ridges; C^2^Discontinuity and epidermal thickening.

Skin appendage: HF, Hair follicles; SG, Sweat glands; LI, Localized in the indurated area; LB, Localized at the border between indurated areas and normal skin; NL, No localization.

Inflammatory cell infiltration: SD, Located in the superficial dermis; SD&SA, Located in the superficial dermis and surrounding skin appendages.

### Localization of Langerhans cells and nerve fibres in keloid tissues

**Fig. 3:**
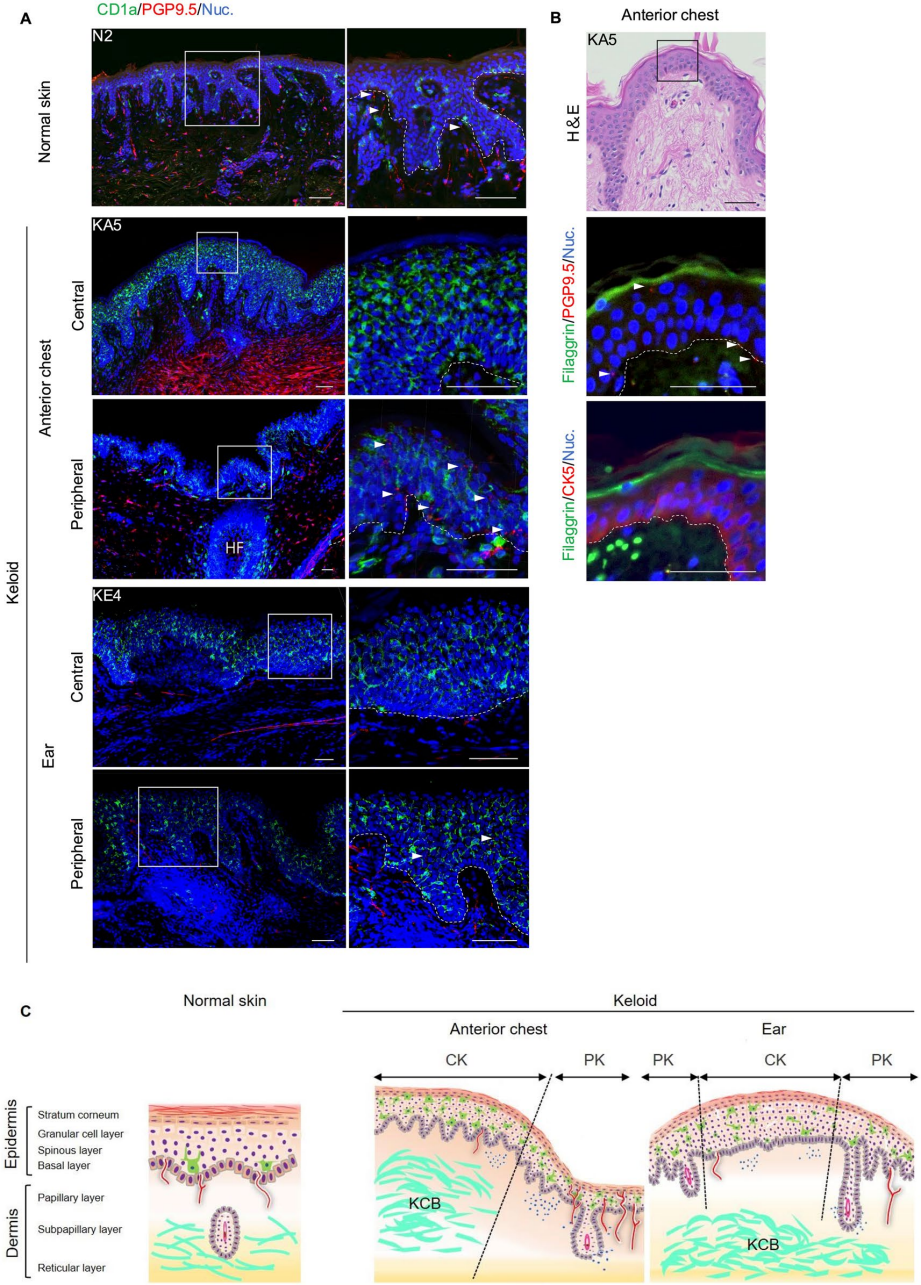
Spatial localization of Langerhans cells and nerve fibres in keloid tissues. (**A**) Immunohistochemical staining of CD1a and PGP9.5 in normal skin (first row from top), anterior chest keloids (second and third rows from top), and ear keloids (fourth and fifth rows from top). The keloids are shown as central and peripheral regions, respectively. Low magnification images of each tissue (left) and high magnification images of the box region (right) are shown. The dashed line indicates the basal layer. Scale bars, 100 μm. HF, Hair Follicles. (**B**) Immunohistochemical staining of filaggrin, PGP9.5 and CK5 in anterior chest keloid. Dotted line indicates basal layer. Red dots (white arrowheads) in the middle image indicate cells that stained positive for anti-PGP9.5 antibody. Scale bars, 50 μm. (**C**) Schematic representation of the localization of intraepidermal nerve fibres and Langerhans cells in keloids and normal skin. The black dotted line is the boundary in the regional classification of keloids. CK, Central region of the keloid; PK, Peripheral region of the keloid; KCB, Keloid collagen bundles.

In inflammatory skin diseases, such as atopic dermatitis (AD) and psoriasis, nerve fibres and Langerhans cells are involved in pruritus. Therefore, we investigated which cells were involved in keloid-associated pruritus using fluorescent immunohistochemistry. CD1a-positive Langerhans cells were localised close to the basal layer of the epidermis in the normal skin (Fig. 3A, N2 and Supplementary Fig. S2); however, in both keloid types, they were broadly distributed within the epidermis, with an increased number of cells observed (Fig. 3A, KA5 and KE4 and Supplementary Fig. S2). In contrast, PGP9.5-positive nerve fibres were focally attached to the basal cell layer in the normal skin (Fig. 3A, N2 and Supplementary Fig. S2), whereas numerous nerve fibres extended into the epidermis, with some reaching the granular layer in the peripheral regions of the anterior chest keloids (Fig. 3A, KA5 lower panel, Supplementary Fig. S2 and movie S1).

We performed immunohistochemical staining using anti-Filaggrin, anti-Protein Gene Product 9.5 (anti-PGP9.5), and anti-Cytokeratin 5 antibodies to analyse the nerve fibres extension into the epidermis in the peripheral regions of the anterior chest keloids. The nerve fibres penetrated the basal layer of the epidermis and extended to the granular layer (Fig. 3B, middle panel). A schematic diagram of each tissue is presented using these results (Fig. 3C). These results imply potential regional differences in the distribution of pruritus-related cells between the keloid types and distinct areas within keloid tissue.

### Quantitative analysis of intraepidermal Langerhans cells and nerve fibre density in keloids

**Fig. 4:**
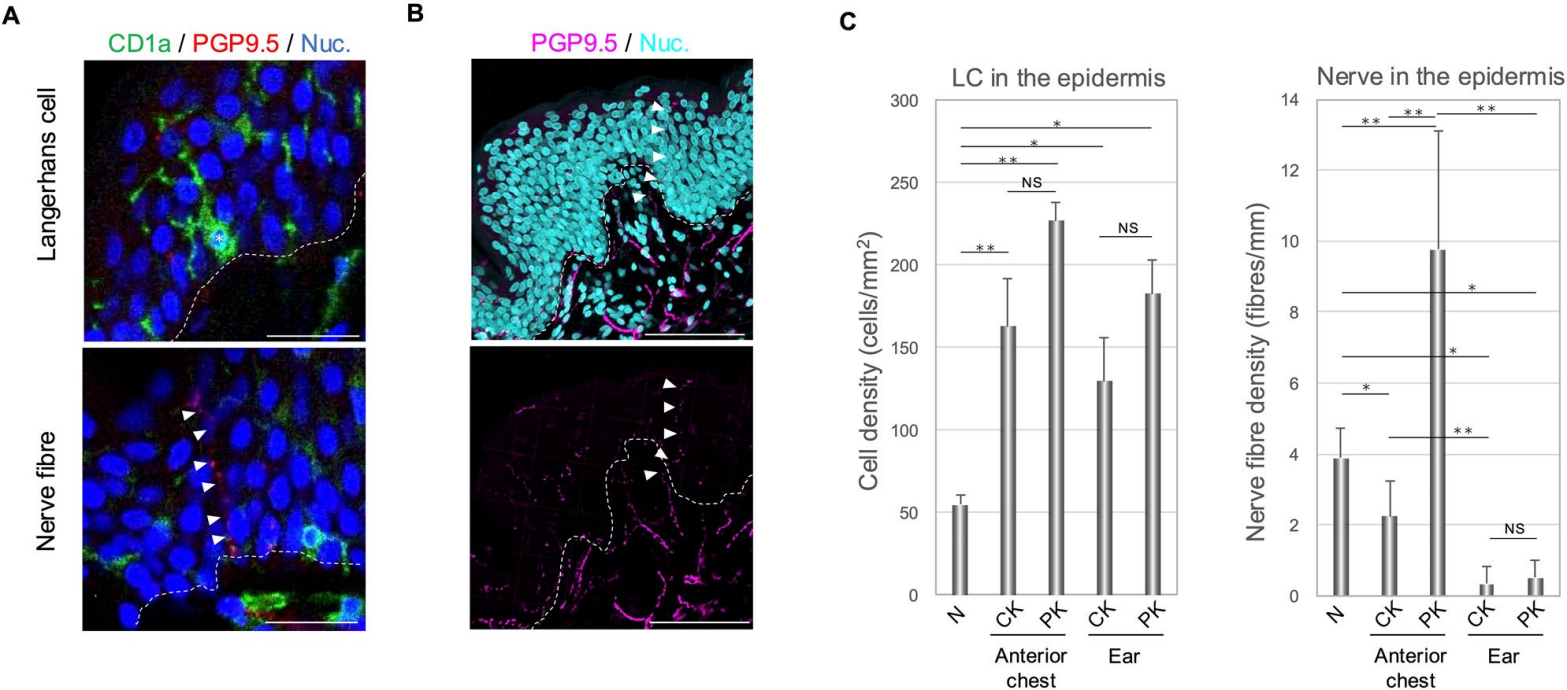
Quantitative analysis of Langerhans cells and nerve fibres in the keloid epidermis. (**A**) Confocal imaging of immunohistochemical staining of CD1a and PGP9.5 in anterior chest keloid. Asterisk indicates the nuclei of the CD1a-positive cells.　White arrowheads indicate PGP9.5-positive nerve fibres. Dashed lines indicate the basal layers. Scale bars, 100 μm. (**B**) Z-stack confocal imaging of Imaris 3D rendering of PGP9.5 positive nerve fibres in anterior chest keloids. Pink dots (white arrowheads) indicate PGP9.5-positive nerve fibres. Dashed lines indicate the basal layers. Scale bars, 100 μm. (**C**) Quantitative analysis of cells showing CD1a and PGP9.5 positivity for epidermal area; *P < 0.05, **P < 0.01 by *t*-test; error bars represent standard deviation.

It is thought that pruritus arises from sensory nerves responding to external stimuli, such as physical and chemical triggers, and pruritus mediators transmitting the sensation. We quantified immunofluorescent-stained cells within thick sections three-dimensionally to elucidate the distribution of Langerhans cells and nerve fibres in keloid tissues. Langerhans cells were counted as individual cells with stained cytoplasm surrounding the nucleus (Fig. 4A, upper panel) and nerve fibres as single units when stained continuously in a fibrous manner (Fig. 4A, arrowheads in lower panel). Nerve fibres extending from the basal layer into the epidermis and reaching the granular layer were observed in the thick sections of the anterior chest keloids (Fig. 4B, arrowheads).

A significant increase in the density of Langerhans cells was observed in both the anterior chest and ear keloids (*n* = 3, 3, and 3 in the anterior chest, ear, and normal skin, respectively), and both the central and peripheral regions, compared to normal skin. However, we did not observe significant differences between different keloid sites or among regions within the same specimens (Fig. 4C). Conversely, the number of nerve fibres that penetrated the epidermis was significantly higher in the peripheral region of 1the anterior chest keloids than in the normal skin and ear keloids (*n* = 3, 3, and 3 in the anterior chest, ear, and normal skin; Fig. 4C). Furthermore, in anterior chest keloids, both the central and peripheral regions exhibited abnormal nerve fibre distribution. In contrast, ear keloids showed a significantly lower number of nerve fibres in both the central and peripheral regions compared to the normal skin and anterior chest keloids (Fig. 4C). In addition, ear keloids did not exhibit the same distribution abnormalities of nerve fibres in the central and peripheral regions as those observed in the anterior chest keloids (Fig. 4C). These quantitative results indicated that pruritus-receiving nerve fibres are distributed in a large number of the periphery of anterior chest keloids, extending up to the superficial layers of the epithelium, indicating an association with the clinical findings of intense pruritus in these regions.

### Expression of pruritus mediators in keloid tissues

**Fig. 5:**
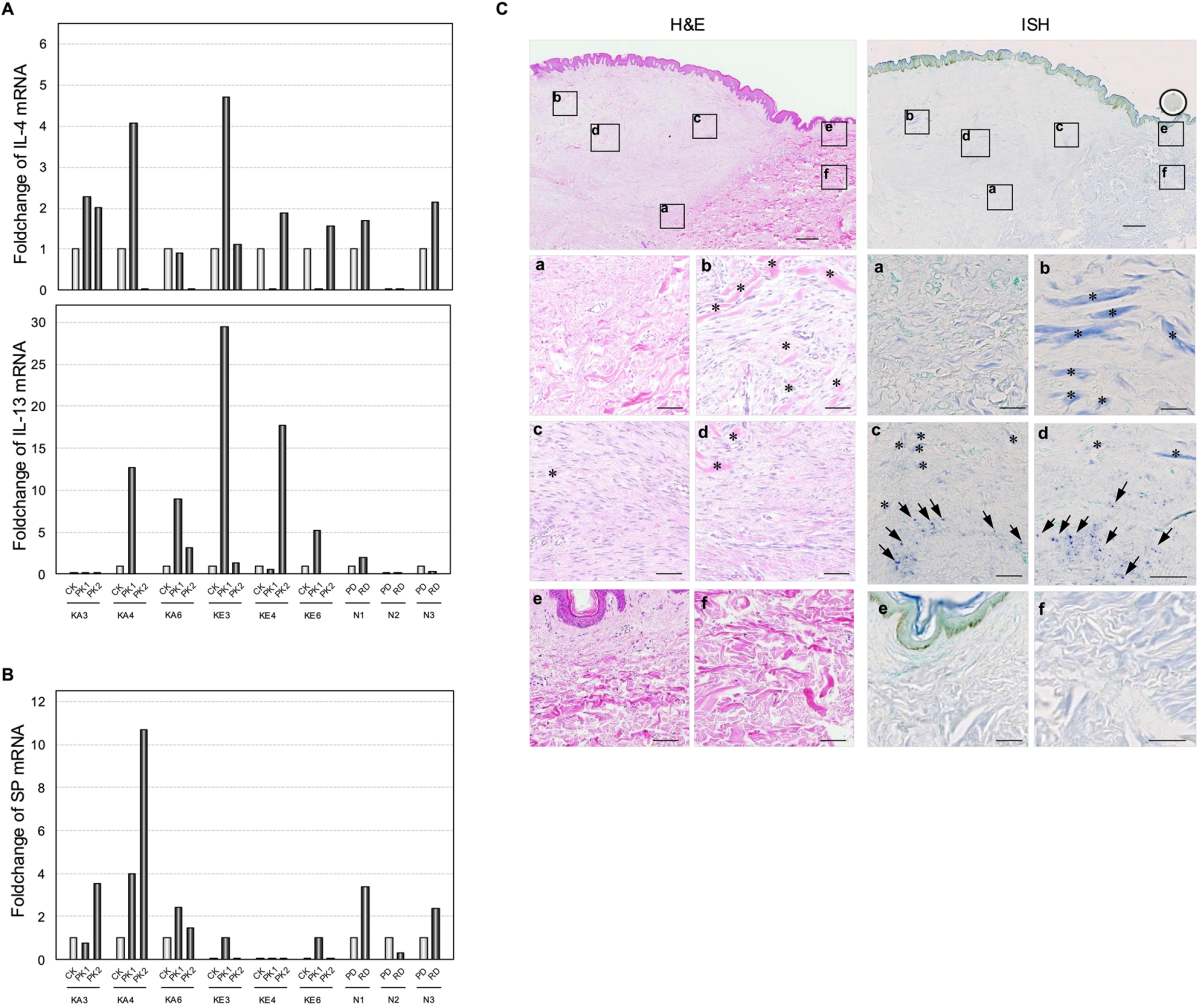
Pruritus mediators in keloids. (**A**) Fold change IL-4 and IL-13 expression in specimens. (**B**) Fold change SP expression in specimens. The increase or decrease in the expression level of Th2 cytokines and SP in the peripheral region of keloid relative to that in the central keloid region in the same specimen is shown. In normal tissues, the relative these molecules expression levels were compared between papillary dermis (PD) and reticular dermis (RD). N, Normal skin; KA, Anterior chest keloids; KE, Ear keloids. CK, Central region of the keloid; PK, Peripheral region of the keloid. The light gray represents CK and PD, while the dark gray represents PK and RD in A and B. (**C**) H&E staining of anterior thoracic keloid tissue. (Right Panel) In situ hybridization of the substance P gene in the anterior thoracic keloids. High magnification images of each box area are shown below the low magnification image. Alphabets in the boxes of the low magnification images correspond to the high magnification images. The thick blue heterochromatic band (asterisk) indicates keloidal collagen. Blue dots (arrows) indicate positive signals. Scale bars, 100 μm.

Diverse pruritus mediators are recognised as potential therapeutic targets. Therefore, it is reasonable to assume that these mediators may be present in or near the areas where pruritus occurs in keloids. In anterior chest keloids, we observed that both the central and peripheral regions exhibited abnormal nerve fibre distribution (Fig. 4C). To investigate pruritus mediators in keloids, we analysed the Th2 cytokines, IL-4, IL-13, and IL-31, and SP neuropeptide gene expression in anterior chest keloids, ear keloids, and normal skin using real-time PCR. Expression levels of IL-4 and IL-13 tended to be higher in the peripheral regions compared to the central regions of both anterior chest and auricular keloids (Fig. 5A and Supplementary Fig. S3). Similarly, SP gene expression exhibited a peripheral predominance, with elevated levels observed in the peripheral areas of anterior chest keloids (Fig. 5B and Supplementary Fig. S3). In contrast, only a few ear keloids demonstrated detectable SP expression. SP expression in normal skin was lower than in anterior chest keloids, with distinct difference observed between the papillary and reticular layers. In this study. IL-31 expression was not detected in either keloid or normal skin. To further investigate the regional distribution of SP gene expression in precordial keloids, in situ hybridisation (ISH) was conducted. In anterior chest keloids with pruritus, SP gene expression was observed around the keloid collagen (Fig. 5C, right panels c and d). However, this expression was not detected in the central keloid collagen or in normal skin adjacent to the lesion (Fig. 5C, right panels a, b, e, and f). These findings indicate that the peripheral region of anterior chest keloids with pruritus shows lymphocytic infiltration and increased intraepidermal nerve fibres, along with elevated expression of pruritus-related mediators such as IL-4, IL-13, and SP.

## Discussion

In this study, the three-dimensional analysis of keloid tissue revealed an increase in the nerve fibre density within the epidermis of the pruritic regions, along with the extension of these fibres to the epidermal granular layer. These observations suggest that the localization of nerve fibres within the epidermis could contribute to keloid-associated pruritus. Although the influence of different nerve innervation in each site, medical history, and treatment history cannot be denied, this study demonstrated for the first time a correlation between the distribution of epidermal nerve fibres and pruritus symptoms in different keloid sites and types of pruritus.

Reportedly, in inflammatory skin diseases, including AD and psoriasis, which present with intense pruritus, the intraepidermal nerve fibre density increases compared to that in the normal skin^20,31–33^. An increase in nerve fibre density may be associated with enhanced peripheral sensitivity to pruritus, which is mediated by receptor upregulation that responds to various stimuli from skin cells, including immune cells and keratinocytes^18^. Furthermore, it has been reported that in AD, a disruption of the pruning mechanism of nerve fibres at tight junctions during epidermal turnover may cause abnormal activation of epidermal nerves and uncontrollable pruritus^34^. It has been suggested that a slight decrease in the number of epidermal Langerhans cells associated with skin inflammation leads to monocyte recruitment from the bloodstream that differentiates into Langerhans cells in the epidermis^35,36^. In addition, an increase in the number of Langerhans cells may promote a Th2-dominant state and contribute to the development of AD^25–27^. Th2 cytokines produced by Th2 cells and comprising IL-31, IL-4, and IL-13, which elevated expression in AD, act on nerve fibre receptors and induce pruritus^37^. Our findings indicate that epidermal Langerhans cells are widely distributed and increased in number in keloids. In addition, increased expression of IL-4 and IL-13 was observed in the peripheral regions of keloids. In particular, in anterior chest keloids, pruritus was observed in the peripheral regions, and increased expression of Th2 cytokines and increased nerve fibre density were also confirmed in the same areas. These results suggest that increased epidermal Langerhans cells may promote Th2 immune responses and thus indirectly contribute to pruritus. Considering these findings, these findings suggest that one of the main factors behind the uncontrolled pruritus in keloids is the effect of pruritus mediators such as Th2 cytokines on nerve fibres.

Previous studies have reported that IL-4 and IL-13 act directly on human sensory nerves to increase the expression of neuropeptides such as SP in addition to enhancing excitability^38–40^. SP affects various cells and induces vasodilation, increases vascular permeability, mast cell degranulation, flare reactions mediated by axonal reflexes, regulates inflammatory cytokine release, and mobilises inflammatory cells through increased blood flow^41^. Neurogenic inflammation, in which SP contributes as a pruritic mediator, is mediated by the release of SP from sensory nerve endings. This process stimulates specific receptors on dermal mast cells, leading to the release of histamine, which induces pruritus by acting directly on receptors present on other sensory nerve fibres^33,41–45^. Neurogenic inflammation has been implicated in the pathology of inflammatory skin diseases and has become a treatment target for the development of therapeutic agents against intractable pruritus^6,21–24^. In this study, we revealed for the first time that SP, known as a pruritic mediator, is expressed in the periphery of anterior chest keloids, particularly in the reticular dermis near the keloid collagen at the boundary. A significant increase in the density of epidermal nerve fibres and expression of SP was observed in the periphery of the anterior chest keloids, indicating a favourable environment for the onset of neurogenic inflammation. These findings point to a potential involvement of neurogenic inflammation in keloid-associated pruritus. It is likely that the neurokinin-1 receptor antagonists, which have shown effectiveness in treating chronic pruritus, could potentially offer a therapeutic option for patients with keloids.

In previous studies on keloids, their regional characteristics have mainly been evaluated using clinical findings. e.g., the active areas, which are red and raised and extend into the surrounding healthy skin, are the peripheral region, whereas the areas of enhanced redness and elevation compared to the peripheral region are the central region^46–49^. There have been reports of severe pruritic symptoms in the periphery^49^with a high number of fibroblasts during the proliferative phase^46^ and the infiltration of immune cells^11,50^. In contrast, the central region showed a decrease in cellular components and reduced activity^50,51^. However, the definition of local characteristics in keloids varies among studies, and no consensus has been established. In this study, increased numbers of Langerhans cells and nerve fibres, clusters of lymphocytes presumed to secrete Th2 cytokines, and elevated expression of Th2 cytokines and SP were observed in anterior chest keloids with pruritus. All of these findings were observed in regions corresponding to the pruritic areas. In the present study, we included only cases that were eligible for surgical treatment, although, there are limitations, the comparison of clinical findings and tissue analysis of anterior chest and ear keloids revealed the heterogeneity between keloids, further emphasizing the previously reported differences between keloids^52^. There is still no consensus on the most effective treatment for keloids^5^but these results suggest that inhibitors targeting Th2 cytokines and substance P (SP) may be effective for the treatment of pruritus in keloids. Future research should aim to include an adequate sample size and expand the investigation to regions beyond the anterior chest and ear, which may provide more comprehensive insights.

## Materials and methods

### Tissue specimens

This study was reviewed and approved by the Ethics Committee of Kobe City Medical Center General Hospital and Riken in compliance with the Helsinki Declaration. We enrolled six patients with anterior chest and ear keloid, respectively, and three patients who underwent reconstructive surgery between March 2017 and September 2023 in this study at the Department of Plastic and Reconstructive Surgery, Kobe City Medical Center General Hospital, Kobe, Japan (Table 1). Due to ethical considerations and the challenges involved in tissue collection, the number of patients included in this study was somewhat limited. In particular, precordial keloids are known to have a high recurrence rate, and surgical interventions are generally approached with caution^4^. Consequently, we gathered as much data as possible and performed a descriptive analysis. The keloid diagnosis was made based on the clinical appearance, history, and anatomical location. All lesions analysed in this study satisfied the histopathological criteria for keloids. The pruritus scoring was assessed using the evaluation table of JSS (Table 1). Informed consent was obtained from all patients before conducting the study. Three full-thickness normal skin samples were obtained from a patient during reconstructive surgery.

### Histological and immunohistochemical studies

We fixed tissue samples in 4% paraformaldehyde (PFA) at 4°C and embedded them in paraffin or Tissue-Tek Optimal Cutting Temperature Compound (Sakura Finetek Japan Co., Ltd., Tokyo, Japan) for histological analysis. Paraffin Sect. (5 μm) were stained with haematoxylin and eosin and observed using Axio Scan.Z1 (Carl Zeiss, Oberkochen, Germany). Furthermore, we prepared paraffin and frozen Sects. (10 and 50 μm, respectively). We performed immunohistochemical analysis of D45, CD3, CD19, and CD16 on deparaffinised sections after antigen retrieval under each condition (Table 3), blocking endogenous peroxidase and nonspecific protein binding activity. Furthermore, incubation of primary and biotinylated secondary antibodies (detail conditions as shown in Table 3) was conducted using VENTANA BenchMark ULTRA (Roche-Ventana Medical Systems, Tucson, AZ, USA) for 32 min at 36°C. The sections were stained using a 3,3’-Diaminobenzidine substrate kit (Vector Laboratories, Tokyo, Japan) and counterstained with haematoxylin. We blocked 50-µm thick frozen sections in Tris-buffered saline containing 1% bovine serum albumin, 0.5% Triton X-100 (Sigma, St Louis, MO, USA) for 2 h at room temperature, and incubated overnight with the primary antibodies in blocking solution at 4°C. The bound primary antibodies were detected using secondary antibodies in a blocking solution and incubated for 3 h at room temperature. We counterstained the slides with 4 µg/mL of Hoechst 33258 dye (Dojindo, Kumamoto, Japan) during secondary antibody incubation. Details of the primary and secondary antibodies and the associated epitope recovery methods are shown in Table 3. All fluorescence microscopy images were acquired using a Laser Scanning Microscope 780 confocal microscope (Carl Zeiss) and analysed in three dimensions using Imaris (Oxford Instruments, Abingdon-on-Thames, UK).

**Table 3:** Primary and secondary antibodies used for immunohistochemical analyses.

Antibodies	Source	Specimen condition	Cat#	Target retrieval	Dilution
*Primary*					
Anti-CD45 antibody	Abcam	P	ab10558	TRS (pH6)	1/50
Anti-CD3 antibody [SP162]	Abcam	P	ab135372	CA (pH8)	1/150
Anti-CD19 antibody [SP291] - C-terminal	Abcam	P	ab227688	EDTA (pH8)	1/100
Anti-CD16 antibody	Abcam	P	ab203883	-	1/200
Anti-PGP9.5 antibody produced in rabbit	Sigma-Aldrich	P/FR	SAB4503057	TRS (pH6) / -	1/500
Anti-CD1a antibody [C1A/711]	Abcam	FR	ab201337	-	1/500
Anti-filaggrin antibody [FLG/1561]	Abcam	P	ab1218395	TRS (pH6) / -	1/200
Anti-cytokeratin 5 antibody [EP1601Y]	Abcam	P	ab52635	TRS (pH9) / -	1/200
*Secondary*					
Alexa Fluor ^TM^ 488 donkey anti-mouse IgG (H + L)	Thermo Fisher Scientific		A21202		1/500
Alexa Fluor ^TM^ 594 donkey anti-rabbit IgG (H + L)	Thermo Fisher Scientific		A21207		1/500
Goat anti-rabbit IgG (H + L), biotinylated	Vector Laboratories		BA-1000-1.5		1/200

Specimen condition: P, paraffin-embedded; FR, frozen.

Target retrieval: TRS, Tris buffer; CA, Citric acid.

### Quantitative analysis of Langerhans cells and nerve fibres in the epidermis

We visualised the Langerhans cells and nerve fibres within the epidermis using fluorescent immunological staining and took measurements from three cases, each of the anterior chest and ear keloids and normal abdominal skin, with three sections analysed for each case. The keloids were classified into two regions: the central region, which was the lesion and was characterised by keloidal collagen, and the peripheral region, representing the border area with images showing the destruction of skin appendages and normal skin. The periapical area was defined as the region extending from the skin appendage to the resection margin for ear keloids. Langerhans cells were counted as the stained cells surrounding the nuclei, whereas nerve fibres were counted as one continuously stained fibre. We quantified density of Langerhans cells and nerve fibres by determining the epidermis area and length, respectively, using the ImageJ measurement function^53^.

### Real-time PCR

RNA was extracted using the ReliaPrep FFPE Total RNA Miniprep System (Promega) and reverse-transcribed using the SuperScript VILO cDNA Synthesis Kit (Thermo Fisher Scientific). Real-time PCR was performed using the TaqMan Gene Expression Assay system (Thermo Fisher Scientific) on an Applied Biosystems QuantStudio 12 K Flex instrument (Thermo Fisher Scientific). Gene expression levels were normalized to ACTIN as an internal control. The primer pairs used for real-time PCR are listed in Table 4.

**Table 4:** Primers used in this study.

Gene	Source	Cat#
ACTIN	Thermo fisher scientific	Hs01060665_g1
tachykinin precursor 1 (SP)	Thermo fisher scientific	Hs00243225_m1
interleukin 4	Thermo fisher scientific	Hs00174122_m1
interleukin 13	Thermo fisher scientific	Hs00174379_m1
interleukin 31	Thermo fisher scientific	Hs01098710_m1

List of primers used for real-time PCR.

### In situ hybridization

We prepared a Digoxigenin (DIG)-labelled RNA probe for SP-specific messenger-RNA using the DIG-RNA labelling kit (Sigma-Aldrich, MO, USA). SP-specific primers were designed using published sequence data (NCBI, Gene ID #6863) as an SP forward, AGGAACCAGAGAAACTCAGCACC, and an SP reverse primer, AAAGCACAAGACTGTCAGGAGTTTCC, respectively. We performed the In situ hybridisations using 10 μm frozen sections on coated slide glasses (Superfrost Plus, ThermoFisher Scientific, MA, USA). Specimens were treated with 2 µg/ml Proteinase K (Roche) for 10 min at 37°C, followed by postfixation in 4% PFA in phosphate buffer (19 mM NaH2PO4, 81 mM Na2HPO4) for 10 min at room temperature. Hybridisation was conducted after prehybridisation in a solution consisting of 50% formamide, 5X Saline-Sodium Citrate buffer, 1X Denhardt’s solution, 10 mM EDTA, 0.1% Tween20, and 50 µg/ml transfer-RNA, containing 60 ng/ml DIG-labelled RNA probe at 50°C overnight. Following several wash steps, specimens were incubated overnight at 4°C with an alkaline phosphatase-conjugated anti-DIG antibody. Signals were developed in a Sodium Chloride, Tris-HCl, Magnesium Chloride, and Tween 20 solution (100 mM NaCl, 100 mM Tris–HCl [pH 9.5], 50 mM MgCl_2_, 0.1% Tween 20, and 2 mM levamisole in 12% poly[vinyl] alcohol) containing Nitro blue tetrazolium/5-Bromo-4-chloro-3-indolyl phosphate (Roche) solution. Methyl green pyronine was used for nuclear staining after colour development.

### Statistics and reproducibility

We used Microsoft Excel (Microsoft, Redmond, WA, USA) to conduct statistical analysis using Student’s *t*-test. Values measured for each of the three sections per case were expressed as mean ± standard deviation (SD). Statistical significance was set at *P* < 0.05.

## Electronic supplementary material

Below is the link to the electronic supplementary material.

Supplementary Material 1

Supplementary Material 2

Supplementary Material 3

Supplementary Material 4

Supplementary Material 5

## Data Availability

The specific primer sequences for SP mRNA were designed based on publicly available sequence data from NCBI (Gene ID: 6863; available at: https://www.ncbi.nlm.nih.gov/gene/6863). The datasets created during the study can be obtained upon request by contacting the corresponding author.
