# Supplementary material for: Three-dimensional analysis of intraepidermal nerve fibres and Langerhans cells in keloids with a focus on pruritus
Source: Sci Rep. 2025 Aug 19;15:30313. doi: 10.1038/s41598-025-11674-8 (PMC12365267; doi:10.1038/s41598-025-11674-8)
Supplement: Supplementary file 4 — Supplementary Material 4 [file 41598_2025_11674_MOESM4_ESM.ppt]

## Slide 1
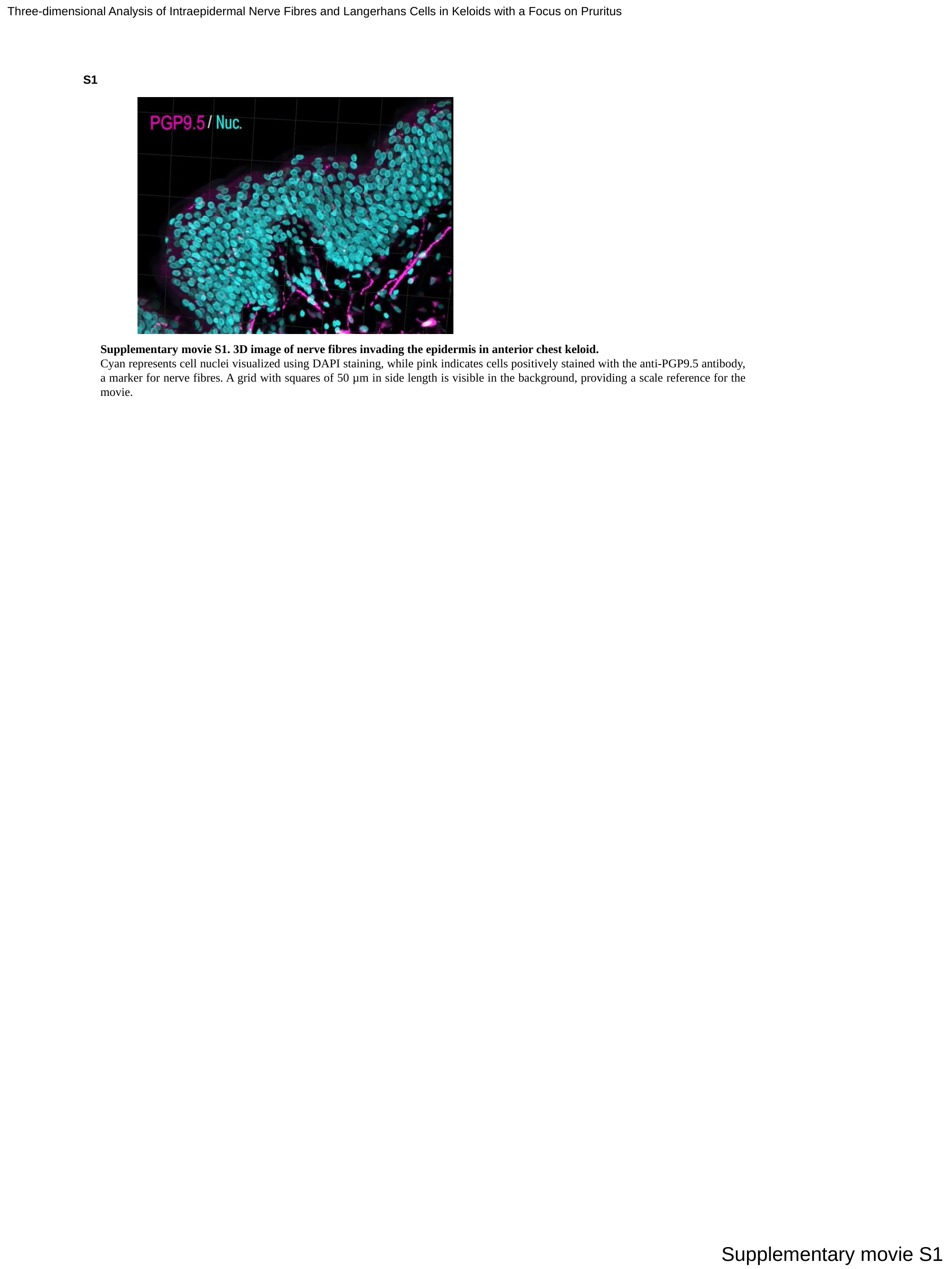

Three-dimensional Analysis of Intraepidermal Nerve Fibres and Langerhans Cells in Keloids with a Focus on Pruritus
S1
Supplementary movie S1. 3D image of nerve fibres invading the epidermis in anterior chest keloid.
Cyan represents cell nuclei visualized using DAPI staining, while pink indicates cells positively stained with the anti-PGP9.5 antibody, a marker for nerve fibres. A grid with squares of 50 µm in side length is visible in the background, providing a scale reference for the movie.
Supplementary movie S1
